# Examining the Mechanisms of Virtual Reality Tourism’s Impact on the Mental Well-Being of Long-Term Care Facility Residents: Perspectives on Presence and Flow

**DOI:** 10.3390/bs14090781

**Published:** 2024-09-06

**Authors:** Yu-Chia Chang, Cheng-Chia Yang

**Affiliations:** 1 Department of Long-Term Care, College of Health and Nursing, National Quemoy University, Kinmen County 892009, Taiwan; ycchang@email.nqu.edu.tw; 2 Department of Healthcare Administration, College of Medical and Health Science, Asia University, Taichung 413305, Taiwan

**Keywords:** virtual reality, tourism, long-term care facility, well-being, presence, flow state

## Abstract

This study investigates the mechanisms of virtual reality (VR) tourism’s impact on the well-being of residents in long-term care facilities (LTCFs). It aims to understand how presence and flow during VR experiences can enhance well-being. This experimental study used a quantitative approach with structured questionnaires to investigate VR experiences among LTCF residents in Taiwan. After obtaining ethical approval, 145 eligible participants from four LTCFs completed a full five-week VR tourism experience. Data collection took place from June to November 2022. This study employed Partial Least Squares Structural Equation Modeling (PLS-SEM) with Smart PLS software to analyze the causal relationships between latent variables. The results confirm that the more vivid the virtual reality image (β = 0.240, *p* < 0.05), the more immersive the experience (β = 0.267, *p* < 0.05), the greater the ability to control the experience (β = 0.465, *p* < 0.001), and the greater the ability to stimulate curiosity during the experience (β = 0.290, *p* < 0.05), the greater the sense of presence. Increased presence leads to user engagement and a state of flow (β = 0.556, *p* < 0.001), which is essential for personal hedonia (β = 0.453, *p* < 0.001) and eudaimonia (β = 0.220, *p* < 0.001). This study elucidates the mechanisms through which VR tourism experiences enhance well-being among LTCF residents, emphasizing the critical roles of presence and flow in promoting both hedonic and eudaimonic dimensions of well-being.

## 1. Introduction

In today’s society, “tourism” is a common way for people to relieve negative emotions (such as stress, fatigue, and depression) and improve their health, quality of life, and well-being [1,2,3,4,5,6]. However, due to physical or cognitive impairment [7,8], long-term care facility (LTCF) residents cannot easily leave their care facilities, let alone participate in tourist activities. Therefore, if technology can surpass these limitations, it may provide a different experience for LTCF residents [6]. Virtual reality (VR) is one way to achieve this goal. VR is a computer-simulated technology that creates a three-dimensional virtual environment and provides users with sensory experiences, such as visual and auditory stimuli, that make them feel as if they are physically present in that environment [9]. With the increasing popularity of VR (such as Google Cardboard) and the increasing abundance of tourist-related VR content, VR technology can remove the distance barrier and change how people travel and experience activities. This type of technology can make it easier for anyone, including LTCF residents, to experience virtual tourism across cities and tourist attractions worldwide [10,11].

VR tourism is attracting increasing attention from researchers and its importance is growing [10,12,13]. Previous systematic reviews have demonstrated the effectiveness of VR for mental health [14,15], and several studies have highlighted the impact of VR tourism on well-being and mental health [16,17,18,19]. For example, McLean et al. used an experimental control design to examine changes in users’ well-being following a VR tourism experience, confirming the positive effects of VR tourism experiences on well-being [17]. Similarly, Aldossary et al. tracked changes in participants’ well-being before and after participating in VR tourism experiences, showing that these experiences can enhance well-being [18]. Although the aforementioned research confirms that VR tourism not only provides users with enjoyable travel experiences but also generates positive psychological effects, the underlying mechanisms of how VR tourism influences well-being remain insufficiently understood [20,21].

Several previous studies have explored partial influencing mechanisms. McLean et al. found that increasing immersion and sensory awareness during VR experiences can help improve VR presence [18], thereby stimulating emotions [22,23,24,25,26]. Presence refers to the degree to which an individual feels immersed in the virtual environment as opposed to their immediate physical surroundings [22]. In addition, Shin pointed out that presence can actively promote flow [27], which is a mental state in which a person is fully immersed in an activity and experiences a sense of focus, engagement, and enjoyment [28]. When users experience a high state of flow, it is easier for them to generate well-being or positive emotional responses [25]. Based on the aforementioned studies that explore the relationship between presence, flow, and well-being, this study will further explore the influencing mechanism more comprehensively.

Through the actual experience of VR tourism by LTCF residents, this study uses VR presence as the core concept to explore the factors that may affect the perception of VR presence and discusses how to influence well-being through a state of flow. The specific research aims include the following: (1) to explore the predisposing factors that influence VR presence; (2) to examine how VR presence can be guided into a state of flow when LTCF residents experience VR tourism; (3) to examine the relationship between state of flow and well-being. The results of this study contribute to a more comprehensive understanding of the process mechanism of how VR tourism experience improves the well-being of LTCF residents.

## 2. Theoretical Basis and Hypotheses

### 2.1. VR and Presence

Based on the functionality of the VR devices, three types can be distinguished (Table 1) [29]: (1) Non-immersive (desktop VR): users interact with the virtual environment displayed on a computer screen using devices such as keyboards, mice, joysticks, or touchscreens; (2) Semi-immersive: this type offers moderately immersive interaction through displays and projection screens, such as Fish Tank VR; (3) Fully immersive: in this system, the user’s environment is completely replaced by the virtual world [30]. This is currently the most immersive VR system and typically requires users to wear head-mounted displays (HMDs) to be fully immersed in the virtual environment. Common devices include the HTC Vive and Meta Quest 2.

VR encompasses several key characteristics, in particular immersion, interactivity, and presence [31]. Immersion refers to the psychological state in which an individual is fully engaged with a virtual environment through continuous sensory stimulation [32]. It can be understood as the objective level of sensory fidelity provided by the VR system and is generally divided into psychological and physiological aspects. Psychological immersion refers to the experiential feelings of the participant, while physiological immersion refers to the sensory stimuli such as auditory and visual cues provided by VR [33]. Interactivity is defined as the interaction between the user and the virtual environment, allowing the user to see the effects of their actions in the virtual space [34]. Presence refers to the user’s sense of being in the virtual environment [35]. Both immersion and interactivity contribute to an increased sense of presence for the user [36].

Psychological research has attempted to explain the reasons behind the role of VR in creating virtual environment simulations. Most research has focused on the concept of presence [15,37,38], suggesting that VR can induce greater presence [39,40]. However, presence is a subjective experience; only the user who has experienced it can judge whether they felt like they were in an actual situation. VR presence depends on a person’s degree of immersion in a virtual environment from the real environment [32]. VR technology helps individuals expand their human senses and cognitive processing system and enhances the vividness and immersion of an image through technology. Thus, it triggers a stronger presence. The current argument regarding VR technology is that presence is the result of immersion [35]. However, presence is more inclusive, expansive, and vital than immersion [41]. In a virtual environment, presence is typically defined as the subjective experience that allows people to exist in a virtual environment through technology [23]. The degree of presence in VR depends on a person’s feelings and the transformation degree from the state of presence in the real environment to immersion in the virtual environment [32]. The theoretical perspective that VR creates presence has been proven to help enhance human behavior. For example, VR presence can help improve the effectiveness of individual attitudes and behaviors [42].

### 2.2. Well-Being

Tourism can indeed effectively improve well-being [1,43]. Well-being is a common mental health measurement variable for the elderly in their later life [44]. It is an overall evaluation of an individual’s life, including emotional reactions to events and judgments of life satisfaction and accomplishments [45]. Therefore, when people experience positive emotions and relationships, actively engage in specific tasks or activities, or feel a sense of accomplishment, they can improve their well-being. Many recent studies have divided well-being into two dimensions [46,47], reflecting that well-being represents a good life [48]. First, “hedonia” can be seen as a concept of seeking pleasure and avoidance. The concept is related to instant sensory pleasure [46]. People seeking this type of well-being will value the well-being of the moment, pursue fun and positive experiences, and avoid negative emotions. Hedonia refers to creating as many positive emotions, such as happiness and satisfaction, as possible within a specific time frame [49]. The second dimension is “eudaimonia”, which includes self-reflection and personal meaning. This concept is related to self-growth and self-realization [50], which suggests that people are committed to pursuing the meaning of life and realizing self-value and self-growth [51]. Eudaimonia is the process of achieving self-realization in a meaningful and more engaging way [52,53]. According to Henderson and Knight [48], some types of activities can only be classified as eudaimonia (e.g., extremely challenging tourist activities) or pure hedonia (e.g., going to an amusement park). However, many tourist activities can still elicit both hedonia (e.g., pleasure) and eudaimonia (i.e., meaning to the individual) experiences. Additionally, applying the above two perspectives to evaluate the concept of well-being will be more complete than using one perspective alone [48,54]; both perspectives are significantly associated with life satisfaction, positive emotions, and well-being [55].

### 2.3. Hypothesis Derivation

Process theory, a commonly used theory in behavioral research, explains how events “happen” and argues that events are specific outcomes that follow a series of certain processes [56]. This theory proposes that the process by which a person’s behavior starts, continues, and stops are related to the motivation and the process that cause the behavior. This theory aims to identify the ultimate key outcome of behavior. It can also help to understand what factors influence people’s behavioral motives. In VR research, recent studies have used the process theory to discuss the impact of users’ behavioral willingness to use VR [57,58]. Different from past research, the present study applied the process theory to conduct a more comprehensive discussion. First, the impact of VR tourism system function and experience quality on presence (input) was examined. We also investigated the relationship between presence and the state of flow (process). Finally, the impact of the state of flow on emotional response (outcome) is discussed.

#### 2.3.1. Factors Influencing Virtual Reality Presence

Bystrom et al. [59] found that the technical characteristics of VR systems or HMD-VR affect presence. VR presence can be improved by enhancing a user’s sense of immersion and sensory perception. Therefore, research suggests that the user’s presence may be affected by the “system function quality” and “experience quality” of VR [58].

Kounavis et al. [60] summarized the factors affecting the function quality of a VR system as the system’s efficacy, efficiency, effectiveness, and vividness. Efficacy can be used to measure whether a VR system is operating normally. Efficiency is used to evaluate whether the VR system’s technology and equipment are running smoothly. Effectiveness is used to evaluate whether a VR system can improve the user’s experience. Vividness emphasizes the stimulation and influence elicited from system pictures or images and focuses on the intensity and clarity of an individual’s impression of the system. These concepts have been proven to help evaluate the technical quality of VR tourism [58].

However, efficacy and efficiency regarding the function quality of VR systems identified by Kounavis et al. [60] are biased toward the technical level; they have been primarily used to evaluate whether the technology and equipment of VR systems can operate normally, along with its convenience. The present study used VR videos as experimental materials. Therefore, only two factors, namely “effectiveness” and “vividness,” were selected to measure the function quality of the VR system. This study suggests that, when LTCF residents experience tourism VR, if they can feel the lifelike and attractive VR video and sound effects (i.e., effectiveness), and the VR video or images are clear and precise (i.e., vividness), the users will be more immersed in the virtual environment and the presence in VR will be strengthened. The following hypothesis is proposed accordingly:

**H1.**  *System function quality (including (a) effectiveness and (b) vividness) positively impacts presence.*

Another key prerequisite for VR presence is “experience quality”, which we discuss using the cognitive absorption theory [61]. Cognitive absorption is a state of interaction between users and software and information technology, i.e., the process of an individual’s full engagement in and use of software, games, or technologies. It describes a deep interaction between people and information technology comprising five main dimensions [61], including the following: (1) Temporal dissociation is defined as the state in which an individual does not feel the passage or delay of time when interacting with information technology; (2) Focused immersion refers to full participation in an activity without interference from unrelated external factors; (3) Heightened enjoyment describes the enjoyment elicited by people participating in an activity and can also represent the feelings from good interactions between an individual and activity; (4) Curiosity, such as the cognitive and sensory curiosity caused by images or VR technology; (5) Control, which indicates that an individual is entirely in control of their interaction with an activity.

Recently, cognitive absorption has been used to understand individuals’ evaluations of virtual technology use [62]. Wei et al. [58] found that VR presence primarily depended on the user’s immersion and interaction degree. Further studies suggest that, the more reality the user feels in a virtual environment, the stronger the immersive feeling is, and the more likely it is that presence is experienced [10,63]. Regarding the ability to control the activity content of VR, control is an essential psychological determinant of VR presence [63]. Therefore, it is reasonable to hypothesize that, when LTCF residents experience VR tourism, if they are immersed in experiencing VR without being aware of the passage of time (i.e., temporal dissociation), this can prevent outside interference (i.e., focused immersion), and users can feel happy and joyful (i.e., heightened enjoyment). Furthermore, they feel that they can control the VR experience (i.e., control). They become more curious and engaged in imagination throughout the experience (e.g., curiosity). Their presence in the virtual environment may be enhanced as a result. Therefore, the following hypothesis is proposed:

**H2.**  *Experience quality (including (a) temporal dissociation, (b) focused immersion, (c) heightened enjoyment, (d) curiosity, and (e) control) positively impacts VR presence.*

#### 2.3.2. Relationship between Presence and a State of Flow in Virtual Reality

VR presence can stimulate emotions [24,25,26,27]. However, the present study proposes that another factor may affect presence and emotional response. This factor may be the state of flow. The concept of a flow state indicates that, when people are fully engaged in the task they are performing, they may focus all of their attention on the activity they are doing at the moment; they may ignore other perceptual information that is not relevant to the task in the process [28]. The state of flow can be an optimal experience (e.g., joyful, positive, happy state) for an individual during work or leisure time.

Weibel and Wissmath [64] found that presence and flow are entirely different concepts. However, there is a close correlation between them. Presence can be defined as the feeling in a virtual world, while flow refers to the feeling of participating in a virtual activity. Therefore, research suggests that presence is often used to describe the degree of immersion in a virtual environment, conceptually focusing on technical characteristics. In contrast, flow refers to the experience of immersion in an activity whose concept is more focused on the characteristics of the task. Several past studies suggest that presence is a prerequisite for flow and that presence has a positive and significant effect on flow [27,64,65]. Therefore, the present study proposes that LTCF residents may experience increased presence when they are immersed in a virtual environment through VR. LTCF residents may be more focused on participating in an activity, and experience increasing presence. As a result, users may be fully integrated into the experience and generate a flow state. Thus, we hypothesize the following:

**H3.**  *VR presence positively affects the state of flow.*

#### 2.3.3. Relationship between the State of Flow and Well-Being

Past research indicates a positive correlation between flow and well-being [66,67]. For example, studies have found that, when tourists acquire a strong flow experience from surfing, they also obtain higher levels of well-being [68]. People who experience a state of flow for a longer period are more likely to display higher levels of well-being than people of the same age [69]. Flow can be seen as a means for individuals to achieve a good life [70], which indicates that flow is closely related to well-being [71,72].

Moreover, the state of flow contributes to active and positive emotions [73]. Csikszentmihalyi [28] found that any activity, whether mental or physical, can generate flow. If people become focused on a task without being affected by external interference, a state of flow may emerge. This can strengthen internal motivation and stimulate positive emotions. Flow is often used to predict enjoyment from leisure activities (such as computer games) [74]. It can predict whether a task is enjoyable for the user based on whether a flow state is achieved. Therefore, people can focus on engaging in various activities to achieve a flow state and obtain positive emotional outcomes, including happiness, concentration, relaxation, confidence, and positive energy [73]. The state of flow is more concerned with the nervous system’s response and physiological processes, and it is closely related to positive emotions, well-being, and life satisfaction [75].

Recent studies indicate that, when users focus on tasks in VR games, they can induce positive emotions and reduce negative emotions [73]. Furthermore, when users complete game tasks, they may have a high sense of well-being, including pleasure and a sense of accomplishment. Research also indicates that, when users focus on participating in glacier adventure experiences, they may generate two types of well-being, including “hedonia” and “eudaimonia” [76]. Therefore, the present study suggests that, when LTCF residents focus on and immerse themselves in the VR game experience through VR technology, they may generate a state of flow through fun and challenges while further receiving higher hedonia and eudaimonia [77,78]. The following hypotheses are proposed accordingly:

**H4.**  *The state of flow positively affects hedonia.*

**H5.**  *The state of flow positively affects eudaimonia.*

## 3. Research Method

### 3.1. Conditions for Research Samples

This study adopted an experimental design. To explore the research aims, we conducted an intervention with participants and performed a quantitative investigation and analysis using a structured questionnaire. After this study was reviewed and approved by the Institutional Review Board of Taichung Jen-Ai Hospital (No: 11027), participants were recruited from four LTCFs in Taiwan. According to the conditions for suitable research samples, adult participants over 20 were included in this study. The exclusion criteria for research subjects were determined through discussions between the research team and LTCF care teams (including doctors, nurses, pharmacists, and care attendants). These medical personnel, who were most familiar with the residents’ medical history and health status, assessed each resident individually to ensure that those who might be at risk from the VR experience were excluded. The exclusion criteria were as follows: (1) People unable to watch VR videos due to visual or hearing impairment; (2) People with open wounds or skin conditions on their face or chronic neck pain or injury that causes safety concerns when wearing a VR HMD; (3) People with a history of severe mental illness (e.g., depression, claustrophobia); (4) People with severe dementia; (5) People suffering from serious diseases (e.g., stroke, Parkinson’s disease); (6) People who could not provide their informed consent to participate.

**Figure 1:**
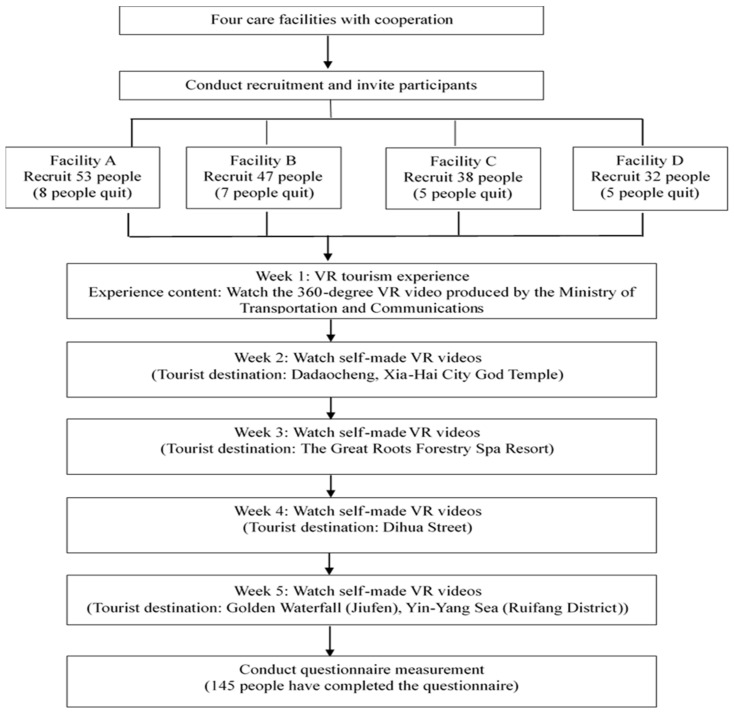
Research Process.

This study used G*Power 3.1.9.7 to calculate the required sample size. According to Faul et al. [79], G* power calculations should have 123 samples under the condition of the statistical power of 80% and medium utility value of 0.15, including 11 predictors. For the present study, researchers personally recruited residents from four LTCFs. After confirming that the potential participants met the requirements for participation, the purpose and experiment content of the study were explained. The study did not begin until the participants’ consent was obtained. Due to the difference in the number of beds available for residents in the four LTCFs, the number of participants eventually included in the study was slightly different (see Figure 1). A total of 170 residents were initially enrolled. However, a total of 25 withdrew from the study. Among them, 20 residents withdrew midway due to illness (including four with dizziness, six with eye problems, and ten with influenza or COVID-19 infection), and five withdrew midway due to personal factors (such as a lack of interest). Finally, a total of 145 LTCF residents completed the full 5-week VR tourism experiment. Chi-squared tests were used to compare dropouts and completers on demographic characteristics (age, gender, educational attainment, and length of stay). The results show that there are no significant differences between the two groups.

### 3.2. Experiment Content and Research Procedure

During the experiment, each facility provided a private space that could accommodate three to five participants at a time for carrying out activities. For safety reasons, the participants were instructed not to stand during the experiment. They were invited to sit or lie down to participate. Researchers assisted the participants in wearing the VR-HMD and started the VR experience once the participants were ready. Throughout the experiment, researchers observed and safeguarded the participants as they experienced the VR simulation. If a participant felt unwell, the experiment was immediately stopped. Participants could also request to watch VR videos repeatedly. All participants were required to complete a VR tourist video experience once a week for five weeks. The video was 3–5 min long (see Table 2). The research samples were collected from 12 June 2022 to 27 November 2022.

The content of the VR tourist experience in the first week of the study consisted of VR videos produced by the Ministry of Transportation and Communications of Taiwan (refer to https://www.youtube.com/watch?v=3ONwddPvob8 (accessed on 12 June 2022)). Next, the VR tourist videos for the remaining four weeks were self-made videos to perform cross-experiences of the urban environment and natural landscapes (see Figure 1).

These VR videos were completed by 360-degree filming and production with Insta360, where three buildings and street attractions in Taiwan were selected for filming in the urban environment, including Dadaocheng, Xia-Hai City God Temple, and Dihua Street in Taipei (refer to https://www.youtube.com/watch?v=x0444 (accessed on 12 June 2022)). For the natural landscapes, three well-known tourist attractions in Taiwan were selected for filming (refer to https://www.youtube.com/watch?v=KKAN0Zmr4CU (accessed on 12 June 2022)), including the Great Roots Forestry Spa Resort, Golden Waterfall (Jiufen), and Yin-Yang Sea (Ruifang District). Regarding the VR-HMD, we adopted Oculus Quest 2, new head-mounted 3D glasses launched by Facebook, for watching VR videos.

### 3.3. Measurement Tools

The questionnaire measurement tools included the self-report method and comprised six parts. The first part involved collecting case data (including age, gender, length of stay, and educational level) and asking the participants about the most impressive attractions or activities in the VR tourist video to ensure data reliability. The second part included “system function quality” and “experience quality”. The evaluation items were developed based on the results of past research regarding information technology and VR system function quality [57,58,61,80]. First, we divided VR “system function quality” into two dimensions, effectiveness and vividness, with eight items. VR “experience quality” contained five dimensions, totaling 18 items. The third part involved collecting the assessment of VR “presence”. We referred to the scale of Vorderer et al. [81] of the conceptualization and operability of presence for subjective measurement, with eight items. The fourth part examined the “state of flow”. According to Kim and Hall [25] and Novak et al. [82], four items were established to measure the flow state. The fifth part was prepared based on the two dimensions of well-being proposed by Lengieza et al. [83]. First, “hedonia” was defined as valuing well-being “in the moment,” pursuing pleasure and positive experiences, and avoiding negative emotions, with six items. We defined “eudaimonia” as the pursuit of life meaning and self-growth experience, emphasizing “long-term” well-being, with six items. A 7-point Likert scale was applied to measure the above items, from 1 (strongly disagree) to 7 (strongly agree).

### 3.4. Data Analysis

This study used the Partial Least Squares (PLS) method for constructing predictive models to analyze the causal model between potential variables. This method is superior to the general linear structure relationship model and is suitable for exploratory research. It can accept a dimension with a single item and is not limited by a variable distribution pattern or the number of samples. Therefore, it has good prediction and interpretation ability [84]. PLS-SEM can detect both path (structural model) and factor (measurement model) in one model. Furthermore, PLS-SEM combines the minimum hypothesis of factor analysis and approximation regression analysis; the resulting R square value represents the degree to which an independent variable can explain a dependent variable. This study used Smart PLS 4.1.0.6 software to analyze the measurement model and structure. We selected 5000 samples using the bootstrap resampling method for parameter calculation and inference estimation [85].

## 4. Results

### 4.1. Descriptive Statistics of Samples

This study included 145 participants for analysis, of whom the majority (90 people, accounting for 62.1%) were women. Most participants (56 people, accounting for 38.6%) lived in LTCFs for less than one year, followed by 46 participants (31.7%) who lived in LTCFs for one to three years. The mean age of all participants was 61.76 years old, with a standard deviation of 6.9. The minimum age was 55, and the maximum was 81. Regarding educational level, most participants (58 people, accounting for 40%) had below primary school education, followed by 47 participants (32.4%) with junior high school education and 40 participants (27.6%) with high school education.

### 4.2. Reliability and Validity of the Research Tools

We used the variance inflation factor (VIF) to ensure no collinearity problem in this study. Table 3 shows that the VIF value of each item was less than 10, and all of them met the standard [86]. Regarding the research model’s fitness, the SRMR of the saturated model and the estimated model in this study was 0.084, which means it had good fitness [87].

Based on Bagozzi and Yi [88], four indicators, namely Cronbach’s alpha, individual item reliability, composite reliability (CR), and average variance extracted (AVE) of potential variables, were adopted to measure the reliability and validity of the research tools. Table 2 shows that the load coefficient of all factors in this study ranged from 0.504 to 0.974, all of which were higher than the recommended value of 0.5 and indicated significance. The Cronbach’s alpha of each variable ranged from 0.832 to 0.963, and the CR values ranged from 0.906 to 0.963, all of which were above the standard of 0.7, indicating good internal consistency of the research model. The AVE value of each potential variable was higher than the standard value of 0.5, which is consistent with Hair et al.’s [89] recommendations. This indicates that the present study has good convergent validity.

Finally, we used the standard that the square root of the average variance extracted (AVE) of potential variables must be greater than the correlation coefficient of other dimensions to measure discriminant validity. The research results show that the square root of the average variance extracted (AVE) was greater than the correlation coefficient of each dimension (see Table 4). This indicates that the dimension has discriminant validity. The above analysis results indicate that each dimension’s reliability, convergent validity, and discriminant validity reached acceptable levels.

### 4.3. Hypothesis Test

**Figure 2:**
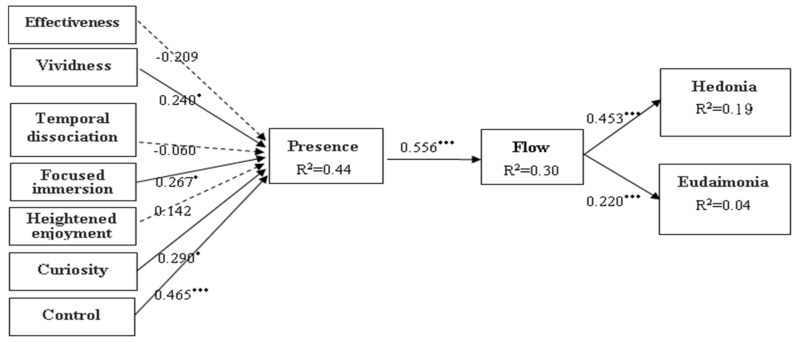
Path diagram of the research hypotheses. *: *p* < 0.05; ***: *p* < 0.001.

We used the bootstrap re-sampling method in PLS to test the significance degree of the paths in the structural model. Among them, R^2^ was the leading indicator to judge the quality of the model [90]. The path relationship between various dimensions was estimated using PLS (see Table 5 and Figure 2). This study examined what factors would affect presence in the VR experience and whether presence would generate a flow state. Finally, we discuss whether a state of flow can affect well-being. The findings show that vividness has a significant difference in presence in the system function quality (β = 0.240, t = 2.150, *p* < 0.05). This indicates that the more vivid the image, the higher the perceived presence. Therefore, Hypothesis H1b is true. However, regarding effectiveness (H1a), there was no significant effect (β = −0.209, t = 1.476, *p* > 0.05). Regarding experience quality, the findings indicate that focused immersion positively affected presence (β = 0.267, t = 2.304, *p* < 0.05). This means that, the higher the immersion elicited by VR, the stronger the perceived presence. Therefore, Hypothesis H2b is true. Curiosity positively affected presence (β = 0.290, t = 2.188, *p* < 0.05), which indicates that, the more the VR experience leads the user to have higher curiosity, the more likely this is to improve presence. Therefore, Hypothesis H2d is true. Similarly, control positively affected presence (β = 0.465, t = 4.059, *p* < 0.001). In other words, in VR experiences, the greater the degree to which the user can perceive and control, the easier it is to generate an immersive presence. Therefore, Hypothesis H2e is true. The above four factors combined account for 44% of the variation regarding presence.

The findings also indicate that VR presence positively affected the flow state (β = 0.556, t = 8.297, *p* < 0.001). In other words, it was easier for the user to generate a state of mind that is focused and fully engaged in the activity itself, along with the increasingly high immersive perception. Therefore, Hypothesis H3 is true and can explain a total of 30.3% variation in the state of flow. The stronger the flow perception, the higher its effect on an individual’s perception of hedonia and eudaimonia (β = 0.453, t = 5.463, *p* < 0.001; β = 0.220, t = 2.574, *p* < 0.001). Therefore, Hypotheses H4 and H5 are also true.

## 5. Discussion and Conclusions

The present study differs from previous research [17,25,58] by adopting process theory [55] to develop a more comprehensive theoretical framework, allowing for a deeper understanding of how VR tourism affects psychological emotions. Our findings indicate that seven of the ten hypotheses were supported. Specifically, this study suggests that the clarity and vividness of VR images significantly enhance the perception of presence. During the VR experience, users can immerse themselves in the task at hand and ignore external distractions (focused immersion), while feeling a sense of control over the activity (control) and becoming more curious and imaginative (curiosity). These factors collectively enhance the sense of presence, thereby increasing the user’s engagement in the experience task, which in turn leads to a state of flow. A higher state of flow is also associated with an increased sense of well-being. This novel perspective highlights how the integration of presence and flow within the VR experience can enhance positive effects on well-being. In addition, our research provides valuable insights into the factors that enhance presence in VR environments, with important implications for the design of VR tourism content. The theoretical and practical implications will be further discussed in depth below.

### 5.1. Theoretical Implications

#### 5.1.1. Predisposing Factors Affecting Virtual Reality Presence

To explore the predisposing factors that influence VR presence, this study found that both system functional quality and experience quality significantly affect users’ virtual experience. For “system function quality”, the sensory properties of a VR system (e.g., vividness) have a significant impact. Van Kerrebroeck et al. [91] found that the vividness of video content positively impacts presence. Wei et al. [58] also found that VR vividness can affect users’ behavioral willingness to use VR; the clearer and more realistic the picture, the greater the sense of presence. Regarding “experience quality”, Makransky and Petersen [36] described that VR presence may be affected by immersion. In other words, presence depends on the user’s perceived immersion. Similar findings were also observed in the present study. The more involved a user feels in the virtual environment, the more the user feels like being in the real situation, and the higher the sense of presence is [58,92]. Compared to immersion, curiosity is a more important factor affecting presence; users with higher curiosity tend to perceive a higher presence in VR [58,93]. Due to the innovative technology of VR, LTCF residents may feel particularly curious when experiencing VR tourism, which further enhances presence. In addition, this study also found that “control” is the strongest predictor of presence during the VR tourism experience of LTCF residents. Past studies have suggested that control is an essential determinant of VR presence [2,35,57]. In the present study, before LTCF residents experienced VR tourism, researchers briefly introduced the tourist attractions involved in the activity, which may have increased their familiarity with these attractions. As a result, participants could control the VR experience and interaction process with a more relaxed and straightforward attitude, further enhancing their sense of integration into the virtual environment.

#### 5.1.2. Relationship between Presence and a State of Flow in Virtual Reality

Our research extends previous findings [17,25,58] by demonstrating how presence and flow in VR experiences enhance well-being. This study integrates these perspectives and shows how the combination of presence and flow in VR experiences can enhance positive effects on well-being. The results indicate that, when people are immersed in VR, it enhances user presence. The higher the presence, the more engaged people are in the activity, allowing users to fully engage in the experience and generate a state of flow [28,64,65,94,95]. Increased immersion in the activity can lead to improved well-being. This is consistent with previous findings that there is a positive relationship between high levels of flow and well-being [25,67,68].

#### 5.1.3. Relationship between the State of Flow and Well-Being

The present study suggests that VR tourism can be instrumental in promoting both hedonia and eudaimonia for LTCF residents. In terms of hedonia, the results are consistent with those of Aldossary and McLean [18], who found that virtual tourism experiences can induce feelings of happiness and joy in users. Regarding eudaimonia, this study reveals a more complex relationship involving flow states and personal growth. Eudaimonia is primarily focused on the experience and engagement of the activity and is more closely related to flow [96]. Research suggests that people may choose challenging tasks to enter a state of flow, which is a mechanism for personal growth [97]. Additionally, flow states from activities are associated with eudaimonia, such as interest, engagement, and enthusiasm [50]. By providing access to diverse virtual environments and activities, VR tourism allows LTCF residents to engage in meaningful experiences that might otherwise be inaccessible to them. These experiences can contribute to a sense of purpose and personal fulfillment, key components of eudaimonic well-being.

### 5.2. Practical Implications

The results of this study can provide practical suggestions and guidance on how to use VR for tourist activities within the long-term care industry. Due to physical or cognitive impairment caused by aging, LTCF residents must often move from their homes to LTCFs, where 24 h care is provided to sustain their living. Therefore, the autonomy, independence, and privacy of LTCF residents may decline as a result. These circumstances can significantly impact LTCF residents’ life satisfaction, physical function, and physical and mental conditions. Research has suggested that these negative conditions can be mitigated through environmental changes or tourism [98]. However, due to the limitations in care and the fact that most LTCF residents have some impairment in physical or cognitive function, it is challenging for them to improve their life quality through tourist activities. Fortunately, recent technological advances have elicited new opportunities for people to use VR tourism as an alternative to actual tourism [99]. The results of the present study confirm that virtual tourism through VR can allow LTCF residents to experience activities they could not participate in previously or to travel virtually to unknown tourist attractions, which can contribute to improving their well-being.

The presence generated by LTCF residents during VR experiences is an essential factor in promoting the generation of flow, and flow is the antecedent factor to creating well-being. Therefore, we recommend that, when promoting their VR services and products, LTCF or VR tourist developers should strengthen the technology and functions that allow users to immerse themselves in the virtual environment to enhance their immersive experience process. As such, users can focus on the task activities and scenes before them, generating a flow state. Through flow, LTCF residents can improve their personal experience and participation in activities while generating joy and well-being [18].

On the other hand, factors that can enhance the user’s presence are also explored during the experience of VR technology. The results indicate that, for VR tourist content, it is essential for users to see vivid, clear pictures. Therefore, we recommend that developers should pay attention to the vividness, fidelity, and sufficient attraction of VR content when designing and promoting their VR products and services. Second, before starting a VR tourist experience, we suggest that relevant staff should conduct a tour introduction for participants, including the history, culture, and points of interest of the tourist attraction. They can even invite participants to share whether they have had a tourist experience at the attraction beforehand. This could enhance the curiosity of users. Then, they may look forward to the VR content they will see and feel more relaxed and connected to the virtual environment, thereby increasing their sense of control and immersion.

### 5.3. Research Limitations and Suggestions

The present study tried to be rigorous but still has some limitations. First, since the tourist experiences of the participants were not assessed in advance, the differences in tourist experiences cannot be compared. Participants who had experienced or visited a particular tourist attraction in the past were more likely to have a higher degree of control during the VR tourist experience, perhaps even generating a greater sense of immersion. However, for participants who had never been to that tourist attraction before, they may have had greater curiosity due to their anticipation of what they would experience. Such differences between the two types of experiences are worth exploring in future research. Second, this study did not discuss the effects of different kinds of tourist activities, in case of failing to distinguish whether the results of this study are caused by the use of the new technology of VR or by the different types of virtual tourism activities it shows [100]. Past research indicates that different types of tourist activities may have different effects on people’s well-being [83]. Other types of activities can meet different motivations and needs for tourism [101] and can lead to different experiences, as well as varying intensity of emotional responses [98]. To further determine the effective intervention measures to improve the mental health of the elderly, verifying the virtual content of VR tourism is an important future research direction.

Third, the participants in this study who underwent the VR tourism experience were selected by the LTCF care team, who are most familiar with the residents, after evaluating their medical history and health status. Consequently, data on each participant’s comorbidities and medication usage were not collected, making further analysis impossible. Future studies are recommended to collect more comprehensive health data with the informed consent of both the LTCFs and the residents. Finally, previous research has indicated that some individuals may experience symptoms such as nausea or dizziness when using VR, commonly referred to as motion sickness [102,103,104]. The study by Munafo et al. even found that the incidence of motion sickness could be as high as 22% to 56% [104]. However, in our study, only 2.3% of participants withdrew due to dizziness. A possible reason for the discrepancy is that the study by Munafo et al. [105] involved participants playing VR games, whereas our study only required LTCF residents to watch VR videos, which did not necessitate continuous movements. This resulted in fewer issues related to postural instability [106] and less severe dizziness. Nevertheless, as Yildirim pointed out, dizziness remains a prevalent human factor issue when using VR [107]. It is recommended that future VR-related research ensures the safety of the experimental process to minimize the risk of adverse reactions.

## Figures and Tables

**Table 1:** Type of VR systems outlined by Mujber et al. [29].

Type of VR Device	Non-Immersive (Desktop VR)	Semi-Immersive	Fully Immersive
Input devices	Mice Keyboards	Joystick Space balls Data gloves	Haptic Gloves HTC Vive Meta Quest
Output devices	Desktop computers Laptops	Large screen monitor	HTC Vive Meta Quest
Resolution	High	High	Medium
Immersion	Low	Medium	High
Interaction	Low	Medium	High
Price	Low	Medium	High

**Table 2:** Activity planning content of the experimental field.

Week	Purpose	Activity Content
1	Watch a 360-degree VR video produced by the Ministry of Transportation and Communications.	Familiar with VR equipment and first experience with 360-degree VR (the length of the video is about 3 min).
2	Watch self-made VR videos (1).	360-degree VR tourism of urban environment (the video length is about 5 min). The tourist destination is Dadaocheng, Xia-Hai City God Temple.
3	Watch self-made VR videos (2).	360-degree VR tourism of natural landscape (the video length is about 4 min). The tourist destination is the Great Roots Forestry Spa Resort.
4	Watch self-made VR videos (3).	360-degree VR tourism of urban environment (the video length is about 4 min). The tourist destination is Dihua Street.
5	Watch self-made VR videos (4).	360-degree VR tourism of natural landscape (the video length is about 5 min). The tourist destination is the Golden Waterfall (Jiufen) Yin-Yang Sea (Ruifang District).

**Table 3:** Reliability and convergent validity of the reflective measurement indicators.

Dimension	Variable	Loading	T-Value	CR	AVE	α Value	VIF
Efficiency	Eff 1	0.880	11.768	0.957	0.842	0.938	3.654
	Eff 2	0.925	14.288				6.013
	Eff 3	0.940	14.557				6.593
	Eff 4	0.924	15.109				4.480
Vividness	Vit 1	0.710	1.641	0.832	0.556	0.873	4.416
	Vit 2	0.899	2.476				3.013
	Vit 3	0.827	5.414				2.762
	Vit 4	0.630	2.293				3.039
Temporal dissociation	Tim1	0.837	8.038	0.867	0.768	0.849	1.912
	Tim2	0.875	8.085				5.883
	Tim3	0.915	9.019				3.945
Focused immersion	Imm1	0.838	16.231	0.888	0.745	0.884	2.219
	Imm2	0.768	8.728				1.583
	Imm3	0.930	43.512				4.024
	Imm4	0.907	35.04				1.912
Fun	Fun 1	0.980	36.005	0.940	0.725	0.924	2.834
	Fun 2	0.974	36.130				2.762
	Fun 3	0.919	23.948				4.514
	Fun 4	0.969	34.489				3.737
Control	Con 1	0.926	23.602	0.925	0.854	0.915	4.024
	Con 2	0.911	28.123				2.507
	Con 3	0.935	37.595				4.221
Curiosity	Cur 1	0.883	39.826	0.917	0.786	0.970	5.480
	Cur 2	0.886	74.402				7.570
	Cur 3	0.892	68.871				7.701
Presence	Pre 1	0.791	13.433	0.932	0.656	0.926	3.163
	Pre 2	0.733	8.357				4.416
	Pre 3	0.800	10.437				3.013
	Pre 4	0.835	15.341				2.762
	Pre 5	0.818	18.418				3.039
	Pre 6	0.858	30.353				3.661
	Pre 7	0.788	13.627				1.563
	Pre 8	0.851	24.488				2.219
Flow	Flow 1	0.716	9.042	0.870	0.716	0.864	1.563
	Flow 2	0.847	21.396				2.219
	Flow 3	0.909	44.973				1.583
	Flow 4	0.898	35.444				4.024
Hedonia	Ple 1	0.864	12.480	0.940	0.725	0.924	5.480
	Ple 2	0.877	29.499				4.638
	Ple 3	0.914	45.752				5.289
	Ple 4	0.833	15.006				7.648
	Ple 5	0.851	24.156				6.502
Eudaimonia	Self 1	0.920	27.133	0.963	0.830	0.950	5.339
	Self 2	0.851	15.828				3.719
	Self 3	0.917	45.747				6.502
	Self 4	0.940	41.522				7.106
	Self 5	0.897	23.754				4.511

**Table 4:** Discriminant validity of the measurement indicators.

	1	2	3	4	5	6	7	8	9	10	11
1	**(0.918)**										
2	0.508	**(0.637)**									
3	0.355	0.329	**(0.876)**								
4	0.433	0.157	0.444	**(0.863)**							
5	0.656	0.395	0.546	0.520	**(0.961)**						
6	0.421	0.298	0.328	0.353	0.430	**(0.924)**					
7	0.611	0.388	0.406	0.435	0.678	0.421	**(0.987)**				
8	0.287	0.351	0.256	0.403	0.318	0.584	0.446	**(0.810)**			
9	0.268	0.339	0.352	0.547	0.373	0.288	0.353	0.556	**(0.846)**		
10	0.617	0.403	0.473	0.495	0.731	0.440	0.630	0.396	0.453	**(0.851)**	
11	0.359	0.450	0.209	0.042	0.289	0.441	0.390	0.494	0.220	0.467	**(0.911)**

Note 1: 1. Efficiency; 2. Vividness; 3. Temporal dissociation; 4. Focused immersion; 5. Fun; 6. Control; 7. Curiosity; 8. Presence; 9. Flow; 10. Hedonia; 11. Eudaimonia. Note 2: Boldfaced figures are the square root values of AVE.

**Table 5:** Results of the research hypotheses.

Hypothesis	Path Relation	Path Coefficient	T-Value	Result
H1a	Effectiveness→presence	−0.209	1.476	False
H1b	Vividness→presence	0.240	2.150 *	True
H2a	Temporal dissociation→presence	−0.060	0.589	False
H2b	Focused immersion→presence	0.267	2.304 *	True
H2c	Heightened enjoyment→presence	0.142	1.380	False
H2d	Curiosity→presence	0.290	2.188 *	True
H2e	Control→presence	0.465	4.059 ***	True
H3	Presence→flow	0.556	8.297 ***	True
H4	Flow→hedonia	0.453	5.463 ***	True
H5	Flow→eudaimonia	0.220	2.574 **	True

*: *p* < 0.05; **: *p* < 0.01; ***: *p* < 0.001.

## Data Availability

The data that support the outcomes of this study are accessible from the corresponding author. Further inquiries can be directed to the corresponding authors.
